# PLLA Membranes Enriched with Chitosan/DCPA: Innovative Approach to Bone Tissue Engineering

**DOI:** 10.4317/jced.61643

**Published:** 2024-07-01

**Authors:** Nader-Yassin Moubarec, Sabrinna-Effgen Pereira, Marco-Antônio Rigo-Rodrigues, Denisse-Esther-Mallaupoma Camarena, Laura-Oliveira Rebouças, Luiz-Henrique Catalani, Maria-Stella Moreira, Leticia-Cristina-Cidreira Boaro, Flávia Gonçalves

**Affiliations:** 1 Departamento de Odontologia. Universidade Ibirapuera. São Paulo (São Paulo), Brazil; 2 Departamento de Odontologia. Universidade de Santo Amaro. São Paulo (São Paulo), Brazil; 3 Departamento de Química Fundamental, Instituto de Química. Universidade de São Paulo. São Paulo (São Paulo), Brazil; 4 Departamento de Estomatologia, Hospital AC Camargo, São Paulo, Brazil; 5 Department of Stomatology, School of Dentistry, University of São Paulo (USP), São Paulo, Brazil; 6 University of Saskatchewan, College of Dentistry. Saskatoon, SK, Canada

## Abstract

Bone tissue engineering has been very promising. The use of scaffolds of synthetic and natural materials is an alternative to combine the advantages of both types of materials. This study aimed to evaluate electrospun polymeric matrices of pure PLLA or associated with 5% or 10% of chitosan particles loaded or not with DCPA in bone proliferation and differentiation of periodontal ligament stem cells (PDLSC). The particles and matrices were characterized by scanning electron microscopy. PDLSC were isolated from periodontal ligament fragments of human permanent teeth using the explant technique. Cell proliferation assay (Alamar Blue) was performed from 1 to 21 days of culture in clonogenic medium and Alizarin Red assay was performed after 21 days of culture in osteogenic medium. The data were analyzed using Kruskal-Wallis test, and the comparison between media was given by the Student-Newman-Keuls test (α = 0.05). On days 1 and 7 there were no statistical difference between materials regarding cell proliferation (p>0.05). The materials with 5 and 10% chitosan / DCPA showed greater proliferation than PLLA control on days 14 and 21 and the material with 10% pure chitosan was greater than the control in 21 days. Regarding the alizarin red assay, PLLA 5% chitosan, PLLA 5 and 10% chitosan / DCPA showed a greater degree of mineralization than the control and the PLLA 10% chitosan material, and they were similar to each other. We conclude that PLLA 5 and 10% chitosan / DCPA materials were able to increase both, cell proliferation and differentiation of PDLSC in bone cells.

** Key words:**Chitosan, PLLA, polymeric matrix, electrospinning.

## Introduction

Bone tissue engineering holds great promise as an alternative for bone regeneration (1). The effectiveness of this therapy hinges on three crucial elements: a scaffold that supports cell adhesion and growth, stem cells that are capable of differentiating into bone tissue, and bioactive molecules guiding the differentiation of these cells (2). However, more research is necessary to find the perfect balance within this trio. An ideal scaffold should: have a mechanically sTable three-dimensional structure; integrate biologically with the implantation site; support the adhesion and growth of host cells; present porosity for metabolic exchanges; and provide the regeneration of the target tissue while as the same time as it degrades (3). Both synthetic and natural materials have been extensively studied to synthetize scaffolds for bone regeneration, each of them offering its own set of advantages and limitations (4,5).

Among synthetic materials, poly-L-lactide acid (PLLA) stands out for its biocompatibility, high mechanical resistance, thermoplasticity, degradation rate partially controllable by molecular weight, and availability in a renewable source (6,7). Chitosan, a natural polysaccharide, has promising properties for bone regeneration. In addition, it is renewable, biocompatible, biodegradable, antibacterial, and non-cytotoxic (8,9). The chitosan could increase alkaline phosphatase activity and cell differentiation of osteoprogenitor stem cells into osteoblasts in a study with rabbits (10). In other side, anhydrous dicalcium phosphate (DCPA), a synthetic inorganic material, exhibits interesting osteoconductive properties for bone regeneration (11). Associated with a biopolymer, it can be a viable alternative to improve biocompatibility and bioactivity results, in addition to being non-toxic (12).

Combining the materials mentioned—PLLA, chitosan, and anhydrous dicalcium phosphate (DCPA)—into a hybrid membrane could be a promising strategy. This approach aims to merge the mechanical strength of PLLA, the biocompatibility of chitosan, and the osteoconductive properties of calcium phosphate, and enhance bone regeneration.

However, electrospinning pure chitosan is quite challenging and typically requires blending with other compounds to be feasible (13). Creating chitosan particles, whether loaded with calcium phosphate or not, is a straightforward and efficient method that our research group has successfully employed (14).

Given the above, this study aimed to synthesize pure chitosan particles or those loaded with DCPA; electrotrophy membranes of pure PLLA and combine with these particles at different concentrations and evaluate their osteoconductive capabilities as well as their support for cell adhesion and growth. The hypothesis posits that PLLA membranes enriched with a higher concentration of DCPA-loaded chitosan particles will more effectively promote the differentiation of periodontal ligament stem cells into bone cells compared to other materials. Additionally, it suggests that membranes with a higher chitosan content will exhibit greater cell adhesion and proliferation.

## Material and Methods

This is an experimental *in vitro* study.

-Synthesis of chitosan particles

The commercial chitosan of high molecular size (Sigma Aldrich, St. Louis, USA) was submitted to a purification process where a 1% chitosan solution was prepared in 3% acetic acid solution. The solution was filtered to remove all insoluble particles and precipitated in a 1M sodium hydroxide solution. The solution was centrifuged at 5000 rpm for 5min, and the process was repeated for two times. The precipitate was submitted to dialysis to completely remove the sodium hydroxide and the chitosan was lyophilized.

Purified chitosan was used do synthetize the particles by electrospray. In this methodology a difference of potential applied between the chitosan solution and collector plate sprays one aerosol of particles in the collector whereas the solvent is evaporated (15). Two types of particles were synthetized: pure chitosan particles (Ch) and chitosan loaded with DCPA (dibasic calcium phosphate anhydrous) particles (DCPA/Ch). For both, 10 mg/mL chitosan were solubilized in a 90% acid acetic aqueous solution. For the last one, also 2.5 mg/mL DCPA was added to the solution. The solution was electrosprayed with the following parameters: flow rate of 0.5 mL/h, 30kV, distance from needle to collector plate of 18 cm, needle of 21G.

-Synthesis of electrospun mats

A solution of 5 wt% PLLA (5 wt%) in chloroform: dimethylformamide (9:1) was prepared, chitosan or DCPA/Chitosan particles were added to the solution at 5 or 10%, according to the experimental group ( Table 1). The solutions were electrospun with the following parameters: flow rate of 3 mL/h, 20kV, distance from needle to collector plate of 18 cm, needle of 21G. After electrospun, the mats were dried in vacuum desiccator and kept dried until use.

-Particles and mats characterization 

The chitosan and DCPA/chitosan particles alone and also the electrospun mats were covered with gold (8nm thickness) and evaluated in scanning electronic microscopy (SEM) with field emission gun, (FEG 7401F, Jeol, Tokio, Japan) at magnification of x 20000 and x2000 respectively. The fibers diameter was measured using the ImageJ software (National Institutes of. Health, Maryland, EUA) with at least 100 measurements for each material.

**Figure 1:**
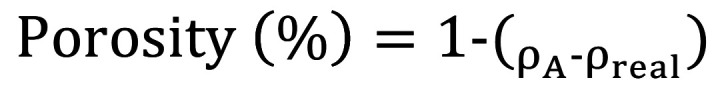
Formula.

The mats porosity was calculated based on the real (ρreal) and apparent density (ρA) of the samples, according to the equation: (Fig. 1).

**Figure 2:**
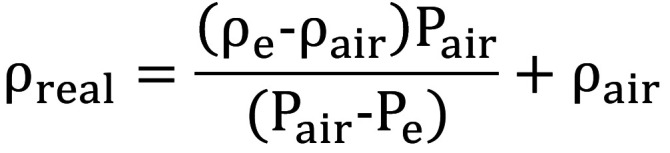
Formula.

The apparent density was calculated by the weight of samples in air (Pair) divided by the volume (area × thickeness) and the real density was calculated, according to the formula bellow, where ρe is the ethanol density, ρair is the air density, Pair is the weight of the samples in air and Pe is the weight of samples in ethanol, (Fig. 2).

-Cells isolation and characterization 

This project was approved by Ethical Committee of Research from University Santo Amaro (CAAE: 59285922.5.0000.0081). Primary cultures from human periodontal ligament stem cells were obtained from fragments of periodontal ligament of two extracted tooth, with surgical indication, using explant technique, after free and enlightened agreement of the patients.

The fragments were kept in culture plates with DMEM/Ham’s F-12 media (Vitrocell, São Paulo, Brasil) supplemented with 15% fetal bovine serum (FBA, Vitrocell). The cells from the explants were debonded by trypsin and placed in new culture plates. Cultures were kept in semi-confluence until use or freezing, to avoid differentiation. The stem cells were characterized immunophenotypically by flow cytometry. Briefly, the cells were washed with PBS and debonded using a using a proteolytic marine enzyme (StemPro Accutase; GIBCO) for 10 min. The cells were centrifuged (550x g for 5 min), resuspended, fixed with 4% paraformaldehyde and marked with primary anitbodies, and, when necessary, they were incubated with secondary antibodies for fluorophore coupling. The follows primary antibodies were used conjugated with allophycocyanin (APC), phycoerythrin (PE), or fluorescein (FITC): CD146-APC (Biolegend, CA, USA), CD90-APC, CD105-FIT (BD Biosciences, CA, USA), STRO-1-FITC, CD44-FITC, CD1-FITC, the uncoupled antibodies CD31-FIT, CD14-PE, and CD45-APC from the manufacturers, and their isotype controls IgG1k-FITC and IgG2k-FITC (all from BD Biosciences, CA, USA). 1x106 cells per surface marker were used and 1x106 stem cells without primary antibodies were considered as the control.

The analysis was performed in a flow cytometer (FACSCalibur, Becton Dickinson, CA, USA), acquiring at least 50,000 events inside the gate and the data were analyzed by FlowJo Software version 9.6.2 (Tree Star, OR, USA).

-In vitro cellular assays

The mats were sterilized 15 min each side under ultraviolet lamp before the *in vitro* cells assay. The cells were culture in high glicose Dulbbecco´s modified Eagle medium (DMEM) supplemented with 10% fetal bovine serum, 100 U/mL penicillin, and 100 µg/mL streptomycin. For the Alizarin Red and alkaline phosphatase assays, 10 mM β-glycerolphoshate, 50ug/mL ascorbic acid and 10-9 M dexamethasone were added to the medium to provide an osteoblastic differentiation medium (ODM).

-Adhesion and proliferation assay

The AlamarBLue Assay was used to measure the cell adhesion and proliferation (n=4). In a 24 wells plate, hPDLSC were placed at concentration of 2×104 cells/well. In 1,7, 14 and 21 days of culture, the medium was removed and 300uL per well, of 10% Alamar Blue in DMEM was added and incubated for 3h in an oven at 37oC and 5% CO2. The absorbance of incubated solutions were read at a spectrophotometer at 570 nm.

-Alkaline Phosphatase assay

The hPDLSC were cultured (1x105 per well) over the mats for 7 days in ODM. The culture medium was removed and the membranes with cells were washed for two times in PBS and immersed in a solution of 1mg/mL p-nitrophenyl phosphate (Sigma Aldrich), 0.05M glycine (Sigma Aldrich) and 2.2 mM MgCl2 (Sigma Aldrich) in water, at pH 10.5 for 15 min. The alkaline phosphatase enzin, broken the p-nitrophenyl phosphate in p-nitrophenol, giving a yellowish color to the solution. The reaction was inhibited, adding NaOH in the same volume. The absorbance of final solution was measured at spectrophotometer at 405 nm.

-Alizarin red assay

The alizarin red assay is an indirect way to measure the cells differentiation, given a measured of calcium deposit in the extracellular matrix, an advanced stage of osteoblastic differentitation.

hPDLSC were added over the mats in a concentration of 1×105 cell/well and cultured for 21 days in ODM. The membranes with the cells were washed with PBS and fixed in solution of 10% formaldehyde in PBS for 10 min.

The fixed membranes were imersed in a 1% alizarin red (Sigma Aldrich) solution with 2% ethanol and stained for 3 min. The membranes were washed abundantly with deionized water and desorvide at 10% cethyl-piridimium chloride solution for 1h. The absorbance of final solution was measured at 570 nm, The mats containing DCPD in their composition were also stained without cells cultured over them and the values were subtracted from the final absorbance after 21 days of the membranes with hPDLSC cultured to discount the initial calcium present in the matrix. The absorbance of final solution was measured at spectrophotometer at 570 nm

-Statistical Analyses

Data were submitted to normality (Shapiro-Wilk test) and homocedasticity test (Levene test) and analyzed by Kruskal-Wallis/Newman test (Proliferation data) and one-way ANOVA/Tukey test (all other tests), with significance global level of 95% (α=0.05).

## Results

**Figure 3:**
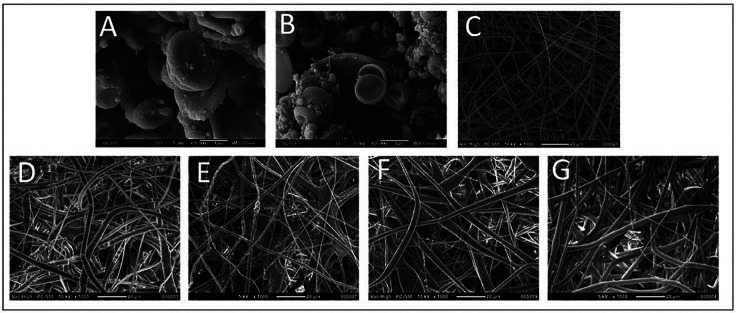
Scanning electronic microscopy of (A) Chitosan particles; (B) chitosan/DCPA particles; (C) PLLA electrospun mats; (D) PLLA + 5% chitosan particles; (E) PLLA + 5% chitosan/DCPA particles; (F) PLLA + 10% chitosan particles: (G) PLLA + 10% chitosan/DCPA particles.

The scanning electronic microscopy images of the chitosan particles loaded or not with DCPA (Fig. 3A,B) presents particles in a spherical shape with mean size of 200 µm. The membranes show random fibers, without drops (Fig. 3C-E), with diameter similar between the PLLA and mats with PLLA_Ch/DCPA_ 5% and PLLA_Ch_10% and statistically greater for the PLLA_Ch_5% and PLLA_Ch/DCPA_10% ( Table 2).

The porosity of the material PLLA_Ch_5% chitosan particles (85 ± 2 %) was similar to the control PLLA group (81 ± 2 %) and statically higher than the other groups which were similar among them ( Table 2) with mean size of 73%.

Cells characterization shows positive expression of mesenchymal and undifferentiated cells: CD90 (99,8%), CD105 (98,9%), CD44 (96,3%), CD146 (60,8%), and STRO-1 (0,67%) and minimal expression for hematopoietic and endothelial cells: CD45 (3,67%), CD14 (2,88%), and CD31 (1,13%).

**Figure 4:**
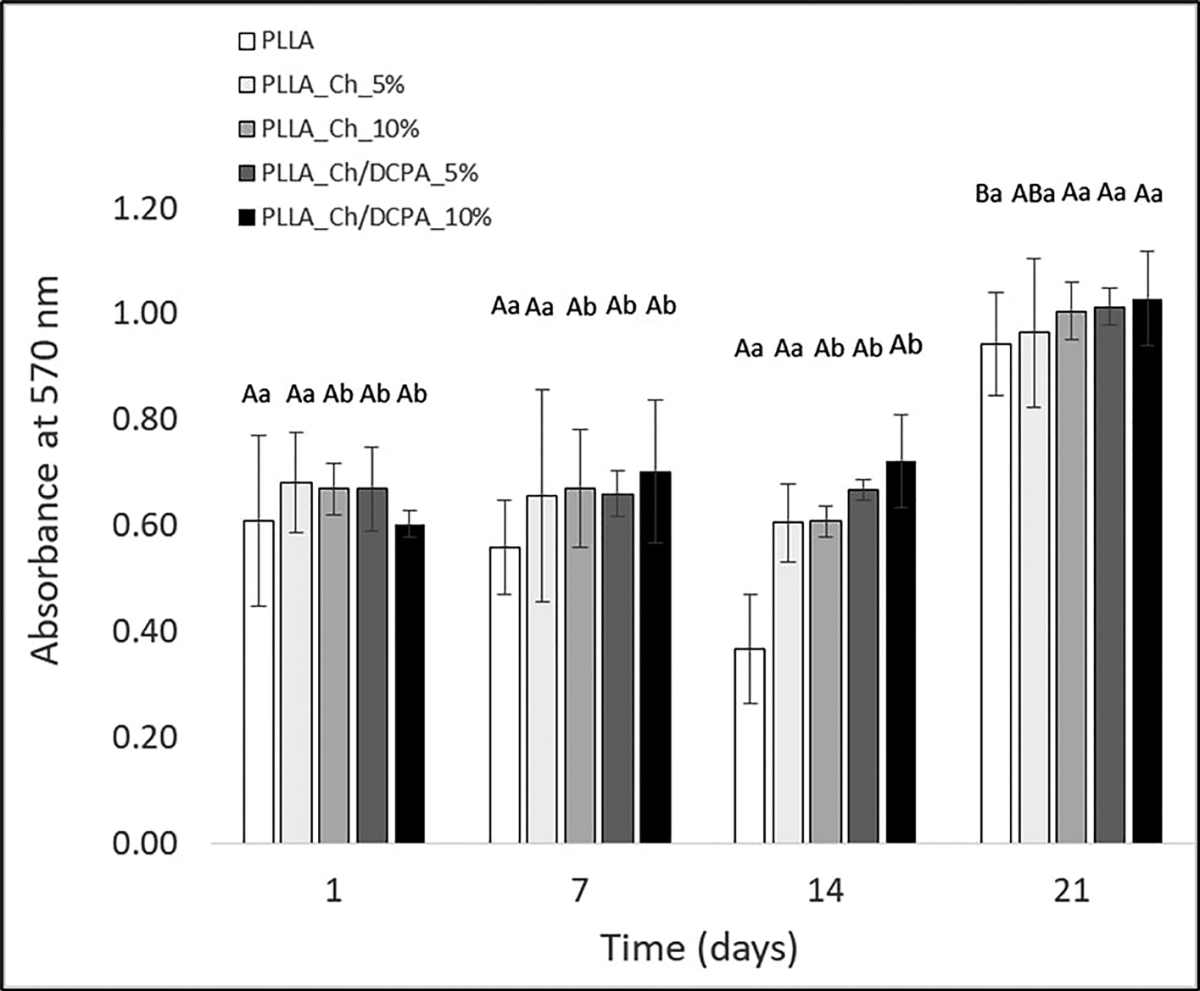
Absorbance of AlamarBlue assay after 1, 7, 14 or 21 days of culture of PDLSC on the membranes. Uppercase letters indicate comparisons of different materials at in the same day and lowercase letters indicate comparisons for the same material at different time.

**Figure 5:**
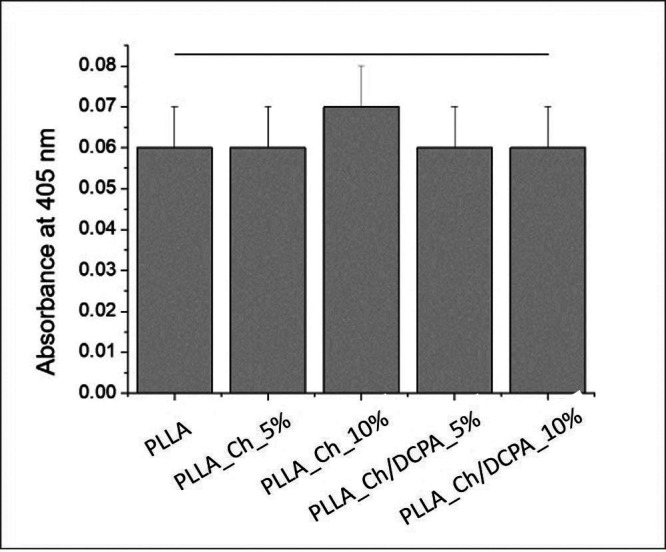
Absorbance relative to the phosphatase alkaline synthese by PDLSC after 7 days in culture on the membranes.Absorbance relative to the phosphatase alkaline synthese by PDLSC after 7 days in culture on the membranes.

The AlamarBlue assay shows the cell adhesion and cell proliferation along the time. There is no difference among the materials regarding hPDLSC proliferation at 1, 7 and 14. At 21 days of culture, the materials PLLA_Ch_10%, PLLA_Ch/DCPA_5% and PLLA_Ch/DCPA_10% showed higher proliferation thatn PLLA or PLLA_Ch_5% (Fig. 4). Also, comparing the same materials along the time, PLLA_Ch_10%, PLLA_Ch/DCPA_5% and PLLA_Ch/DCPA_10% present higher proliferation in 21 days whereas the PLLA and PLLA_Ch_5% present no significant difference on proliferation along the time (Fig. 5).

**Figure 6:**
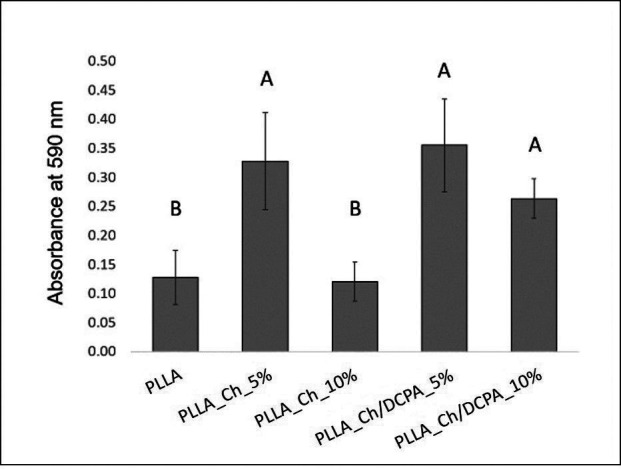
Absorbance of alizarin red assay after 21 days of culture of PDLSC on the membranes.

The alkaline phosphatase assay demonstrates similar synthesis of the enzyme in the materials, at 7 days of culture (Fig. 6). However, alizarin red assay shows significant difference among the materials regarding the extracellular matrix mineralization. The materials containing PLLA_Ch_5% and PLLA_Ch/DCPA_10% showed higher mineralization than the control PLLA or the mat with PLLA_Ch_10% Chitosan particles.

## Discussion

In this study, the synthesis of electrospun mats using the synthetic polymer PLLA, combined with particles of the natural polysaccharide chitosan, which were either loaded with or without dibasic calcium phosphate (DCPA) was performed successfully. This process led to the development of three-dimensional composite membranes with the potential for application in guided bone regeneration and bone engineering.

The electrospinning process resulted mats with randomly fibers, with diameter mean from 1 to 2 µm, high porosity, without presence of beads (Fig. 3), being able to mimetics the extracellular matrix (16). Although the PLLA electrospun is well described in the literature (17), in its pure form or associated different particles (18,19), the electrospun of chitosan is harder (20), and normally associated with addition of other compounds as, polyethylene oxide (21), poly(vinyl) alcohol (22) or poly(ethylene glycol) (23). The electrospinning of PLLA with chitosan particles represents a novel approach. This study not only loads the chitosan particles with DCPA to promote bone regeneration but also demonstrates how the same technique opens up a multitude of opportunities for using chitosan particles as carriers in various drug delivery systems within electrospun mats, enhancing their application potential. Since it can be loaded with different drugs, being an effective carrier when slower released is aimed or in case of hydrophilic drugs, no soluble directly in the hydrophobic solution of PLLA in chloroform.

The hypotheses of this study that higher chitosan concentration increase cell adhesion and proliferation was partially accepted since no differences were observed among the materials until 14 days of culture but at 21 days the control group of PLLA showed lower proliferation than the materials with 10% chitosan or chitosan/DCPA or 5% chitosan/DCPA.

The literature has demonstrated that chitosan facilitates cell adhesion and growth due to its hydrophilic nature and its capability to adsorb proteins with sites for cell adhesion sites (24-26). In the current study, due to its low concentration in the mats (5 and 10%) and because part of the particles was embedded within the fibers, thus not exposed to cell contact, no significant differences in adhesion and proliferation were observed up to the 14th day of culture. In fact, study of Wu *et al*. (27), observed that the addition of 30% chitosan to poly-D-L-Lactideo, results in the increase of proliferation of chondrocytes in relation to control group only after 7 days of culture, considering the differences of concentrations the same behavior can be observed in the present study where the differences observed after 14 days. At 21 days, the materials with 5 and 10% chitosan/DCPA particles and the one with higher concentration of chitosan particles (10%), showed higher proliferation than the control PLLA. At this point, in addition to the effect of chitosan, dose dependent, it can be highlighted the DCPA effect on the proliferation. Several studies have pointed that the addition of calcium ions is associated not only to improvement of cell differentiation but also greater cell proliferation (28-31). Besides of the action mechanism of calcium ions is not elucidated, it is believed due its positive charge, it facilitates the adsorption of proteins from fetal bovine serum, as fibronectin. This is a glycoprotein able to link to integrin, receptors responsible for cell adhesion (28,32), therefore increasing the cell proliferation in the materials which formulations present calcium, in this case DCPA.

The hypothesis that PLLA membranes associated with higher concentrations of chitosan/DCPA particles would exhibit increased hPDLSC differentiation was partially accepted. While no differences in alkaline phosphatase production were observed among the materials, the material containing 10% chitosan/DCPA particles demonstrated higher extracellular matrix mineralization compared to the PLLA group, indicating enhanced bone differentiation at 21 days of culture. Apparently, the effects of chitosan and DCPA are not evident at the begining of cell differentiation (7 days) but it become noticeable in the later stages of bone cell differentiation. This delayed effect can be attributed to the morphology, since the particles are not all at the fiber surface, part of them are inside the fibers, which delays the leaching of ions and the contact of chitosan with the cells.

The presence of functional groups amine (–NH2) and carboxyl (COO-) in chitosan act as crystallization sites which favor the mineral deposit into extracellular matrix (26), enabling some osteoconductive effect to chitosan (33,34). Furtheremore, the osteodifferentiation and osteocontuction potential of the membranes can also be improved by the addition of some calcium phosphates to the scaffolds (24,29,35), as occurs with DCPA (36,37). In the present study, the alizarin red assay evidenced the osteodifferentiation potential of chitosan particles loaded with DCPA, independently of concentration and of chitosan particles without DCPA in low concentration. Curiously, the material with 10% chitosan particles presented result similar to the control group of PLLA whereas the material with 5% chitosan particles presented higher extracellular mineralization. Possibly, this performance result from the charging and dispersion of the particles (38). Particles of pure chitosan are hydrophilic and have higher zeta potential than particles of chitosan loaded with DCPA (14), therefore their dispersion on a hydrophobic PLLA matrix is hard. It is believed, in higher concentrations, higher the particles agglomeration, making their spread into the polymeric fibers, difficult whereas in lower concentrations, grater dispersion can be observed. Similar effect was suggested when chitosan particles where included in a hydrophobic polymeric matrix of dimethacrylates (39), and can justify the lower performance of the PLLA_chitosan_10% material in cell differentiation.

## Conclusions

It can be concluded that the electrospinning of PLLA, when combined with carrier particles of chitosan, whether loaded with DCPA or not, was successful. PLLA mats containing 5 and 10% chitosan/DCPA particles were able to enhance extracellular mineralization and proliferation at 21 days of culture.

## Figures and Tables

**Table 1:** Synthesized electrospun mats.

Material	Composition
PLLA	PLLA
PLLA_Ch_5%	PLLA + 5% chitosan particles
PLLA_Ch_10%	PLLA + 10% chitosan particles
PLLA_Ch/DCPA_5%	PLLA + 5% chitosan particles loaded with DCPA
PLLA_Ch/DCPA_10%	PLLA + 10% chitosan particles loaded with DCPA

**Table 2:** Mean and standard deviation of diameter fiber and porosity of the membranes.

	Fiber diameter (µm)	Porosity (%)
PLLA	1.4 ± 0.2^b^	81 ± 2^a^
PLLA_Ch_5%	2.3 ± 0.8^a^	85 ± 2^a^
PLLA_Ch_10%	1.4 ± 1.3^b^	75 ± 2^b^
PLLA_Ch/DCPA_5%	1.5 ± 0.8^b^	73 ± 2^ b^
PLLA_Ch/DCPA_10%	2.6 ± 1.6^a^	70 ± 4^ b^

## Data Availability

The datasets used and/or analyzed during the current study are available from the corresponding author.
